# First chikungunya outbreak in Pakistan: a trail of viral attacks

**DOI:** 10.1016/j.nmni.2017.05.008

**Published:** 2017-06-01

**Authors:** T.H. Mallhi, Y.H. Khan, A.H. Khan, N. Tanveer, M.I. Qadir

**Affiliations:** 1) Discipline of Clinical Pharmacy, School of Pharmaceutical Sciences, University Sains Malaysia, Penang, Malaysia; 2) Punjab Medical College, Sargodha Road, Faisalabad, Punjab, Pakistan; 3) Institute of Molecular Biology and Biotechnology, Bahauddin Zakariya University, Multan, Pakistan

**Keywords:** Chikungunya, outbreak, Pakistan, *Adese aegypti*, vector borne diseases

## Abstract

Despite explicit warning from the National Institute of Health, Pakistan experienced its first chikungunya outbreak in the metropolis of Karachi. We underscore the attention of health authorities and healthcare professionals towards contributing factors associated with this outbreak and the measures required to combat this viral disease.

In September 2016, the National Institute of Health (NIH) warned Pakistan about the potential risks of chikungunya after its outbreak in India (http://tribune.com.pk/story/1274991/no-preventive-measures-chikungunya-outbreak-traced-back-india/), but Pakistan experienced its first chikungunya outbreak in the metropolis of Karachi, where over 3000 people have been suspectedly infected (http://tribune.com.pk/story/1276248/pakistan-officially-reports-chikungunya-outbreak/).

Pakistan has experienced a quadruple burden of viral infections in 2016, including Crimean-Congo haemorrhagic fever and outbreaks of dengue and chikungunya. These three consecutive viral infections have created much concern among health authorities and the World Health Organization. Epidemiologists linked the outbreak of chikungunya to an outbreak in India a few months before. Although the NIH had issued an alert and also urged the government to keep vigil on cross-border travelling, no preventive measures were taken at airports or railway stations, or at the Pakistan–India border. Pakistan lacks the facilities to screen for viral diseases at airports or at the border, and there is high propensity of disease spreading to other parts of the country (http://tribune.com.pk/story/1274991/no-preventive-measures-chikungunya-outbreak-traced-back-india/). Additionally, heaps of garbage and open, fetid gutters in the city have contributed to the outbreak.

The government drive against chikungunya was, and remains, primarily focussed on spraying for the *Aedes* mosquito vector, but filth and garbage around the buildings, including the provincial government hospital, as well as streets and roadsides were not removed (http://dunyanews.tv/en/Pakistan/366549-Heaps-of-garbage-smelly-gutters-continue-to-spread). The problem lay in the slow response from the provincial government in tackling the outbreak, adding to the misery of poor patients who were deprived of free medicine and proper medical attention at the provincial government hospital. Although a health emergency was imposed in the city, hospitals were closed on public holidays, and patients were thus unable to get their blood tested (http://dunyanews.tv/en/Pakistan/367368-chikungunya-patients-suffer-due-to-closure-of-gove). Unfortunately, entire provinces lack the ability to diagnose chikungunya, so samples were dispatched to the NIH and the United States for identification. Such diagnostic delay and inadequate care of patients can result in human–mosquito virus transmission, which in turn may result in the spread of disease to adjoining areas (https://www.thenews.com.pk/print/173791-chikungunya-epidemic-confirmed).

The history of chikungunya in Pakistan dates to the early 1980s, when Darwish et al. [1] in 1983 detected antibodies against the chikungunya virus from sera of four rodents and one human. However, virus spillover did not occur on that occasion. Later, in 2015, three cases of chikungunya were identified in children during a 2011 dengue outbreak [2]. Despite explicit evidence of the virus in Pakistan, little attention was paid to its monitoring and surveillance, which may be a possible reason for its subsequent disease spread, leading to the current outbreak. Nevertheless, some other factors, including growth-promoting environmental conditions, poor healthcare infrastructure and extensive use of insecticides, can be correlated with this outbreak. In addition to the deplorable sanitary conditions, climate change is also a significant reason behind the reemergence of chikungunya infection in Karachi. Pakistan is ranked seventh among countries that are the most vulnerable to the vagaries of climate change; in Pakistan, summers are getting warmer and winters are getting milder with every passing year (http://www.emro.who.int/pak/programmes/climate-change-and-health.html). These changes in climate provide conditions conducive to the spread of various vector species.

Like dengue epidemics, the chikungunya outbreak is an example of an abrupt expression of vector-borne diseases in the global village. The chikungunya outbreak in India should have served as a warning, but no aggressive action was taken. The government has not taken a hard line, and we believe that putting full effort into controlling the aforementioned contributing factors and providing adequate care would go a long way to combating this outbreak in Pakistan. Chikungunya is self-limiting and has a low mortality rate; however, fatal infections and chronic rheumatic disorders do occur [3]. Pakistan is in dire need of rigorous multidisciplinary efforts to avoid mosquito bites and eliminate mosquito breeding sites. The chikungunya virus can establish itself in any tropical or temperate region with *Aedes* mosquitoes. Thus, key measures for preventing chikungunya epidemics include entomologic surveillance, peridomestic mosquito control, public education, commitment of resources for research, improvements in healthcare infrastructure, detection of imported cases and early recognition of local transmission, followed by efficient vector control [1]. For control of epidemics, vector control is considered to be one of the most important strategies to interrupt or reduce transmission [3]. Vector surveillance and control of the chikungunya outbreak should be approached in an integrated manner.

## Conflict of Interest

None declared.
